# Salvianolic acid A inhibits the activation and aggregation of platelets in patients with acute coronary syndrome

**DOI:** 10.1097/MD.0000000000043305

**Published:** 2025-07-18

**Authors:** Peipei Wang, Shunqiong Zhang, Xinyuan Li, Shuanglin Xie, Yibin Mei, Wei Zhang, Abdullah A.I. Mamun, Wenjuan Lu, Taoqing Liang, Chunlai Zeng

**Affiliations:** a Department of Cardiology, The Lishui Hospital of Wenzhou Medical University, The First Affiliated Hospital of Lishui University, Lishui People’s Hospital, Lishui, Zhejiang Province, China; b Central Laboratory of The Lishui Hospital of Wenzhou Medical University, The First Affiliated Hospital of Lishui University, Lishui People’s Hospital, Lishui, Zhejiang Province, China; c Department of Cardiology, Shaoxing University, Shaoxing, Zhejiang Province, China.

**Keywords:** acute coronary syndrome, CD62p, PAC-1, platelet activation, platelet aggregation rate, salvianolic acid A

## Abstract

**Background:**

The acute blockage in coronary arteries further causes acute coronary syndrome (ACS). There are 2 main factors implicated in the activation and aggregation of platelets. However, this present study aimed to investigate the effect of salvianolic acid A (SAA) on the platelets in patients with ACS and explore its potential mechanism of action.

**Methods:**

The impact of SAA on platelets under different stimulation conditions was studied using flow cytometry and platelet aggregation detection techniques.

**Results:**

The results demonstrated that in 40 ACS patients, ex vivo treatment of platelets with SAA (0.1 mg/ml) significantly reduced aggregation and activation. Intriguingly, we found no significant difference between the 3 types of ACS patients in the antiplatelet effect of SAA. Moreover, the results indicated that C-reactive protein, alanine aminotransferase, C-reactive protein-to-albumin ratio, total cholesterol, and low-density lipoprotein levels were negatively correlated with the anti-platelet effect of SAA in ACS patients and that a history of smoking may reduce the anti-platelet effect of SAA in the same group.

**Conclusion:**

In summary, the above findings of this study highlight the therapeutic potential of SAA against platelets in patients with ACS, providing new insights into clinical treatment and experimental research.

## 1. Introduction

The acute coronary syndrome (ACS) is caused by a blockage of the coronary arteries.^[1]^ However, the blockage may have a variety of consequences depending on its location and extent.^[2]^ Current evidence indicates that cardiovascular disease is the leading cause of death and morbidity worldwide. ACS is usually the first clinical manifestation of cardiovascular disease. A total of 5.8 million new cases of ischemic heart disease were reported in 57 countries that are members of the European Society of Cardiology in 2019.^[3]^ ACS is a cardiovascular emergency involving unstable angina pectoris (UA), non-ST-elevated myocardial infarction (NSTEMI), and ST-elevated myocardial infarction (STEMI), where platelets play a significant role in pathological thrombosis, particularly in coronary arteries under high shear stress.^[4]^ Studies have demonstrated that platelet activation and aggregation further contribute to the development of ACS. The use of antiplatelet therapy is crucial for treating ACS and preventing and treating acute vascular events. COX-1 inhibitors, glycoprotein IIb/IIIa inhibitors, and antagonists of the adenosine diphosphate (ADP) P2Y12 receptor are currently the main approaches for the treatment of ACS.^[5]^ Antiplatelet agents are not effective against large proportions of ACS patients who present “aspirin resistance” and increased P2Y12 receptor activation.^[6]^ Moreover, antiplatelet drugs commonly used in clinical practice can have certain side effects, including reduced reactivity and bleeding.^[5]^ Therefore, the development of effective drugs that inhibit the activation and aggregation of platelets is essential to the prevention and treatment of ACS.

Salvianolic acid A (SAA) is an active ingredient extracted from the Chinese herb Salvia miltiorrhiza that exerts a variety of pharmacological effects such as inhibiting the proliferation and migration of vascular smooth muscle cells, promoting foam cell apoptosis,^[7]^ mitigating the formation and development of atherosclerosis^[8]^ and alleviating myocardial inflammatory responses.^[9]^ Traditional Chinese Medicine promotes blood circulation and removes blood stasis.^[10]^ Several lines of studies have suggested that Salvia miltiorrhiza-related components inhibit the activation and aggregation of platelets, but most of these studies are conducted in animal models, and human studies are lacking.^[11]^ We have found that SAA inhibits the reactivity of platelets in patients with type 2 diabetes mellitus, providing new insight into the solution to antiplatelet therapy in patients' aggregation and activation with atrial fibrillation.^[12]^ Studies have demonstrated that SAA provides promising therapeutic targets by regulating the phosphoinositide 3-kinase-protein kinase B signaling pathway.^[10]^ The mechanism by which SAA reacts with platelets in patients with ACS is still unknown.

The study aims to evaluate the effects of SAA on the aggregation and activation of platelets in patients with ACS and to elucidate the underlying mechanisms, providing new insights for clinical treatments and experimental studies.

## 2. Materials & methods

### 2.1. Participants

The study included 40 patients diagnosed with ACS at the emergency department of Lishui Hospital from October 2022 to February 2023. The following criteria were used to determine inclusion: 18 years or older and consent to sign the informed consent form; diagnosis of ACS; the willingness to participate in the investigation. The following criteria were used as exclusion criteria: hemodynamic instability; severe impairment of liver and kidney function, malignant tumor, human immunodeficiency virus/acquired immune deficiency syndrome; history of blood system diseases, history of diagnosed bleeding diseases, bleeding tendency (such as gastrointestinal bleeding, cerebral hemorrhage, etc); abnormal platelet-related indexes, refusal to sign the consent form. A total of 4 mL of blood was collected from each participant and placed into a silicified vein containing 3.8% sodium citrate in a volume ratio of 1:9. The expression of platelet activation markers and the maximum platelet aggregation were determined using flow cytometry and light transmission aggregation.

The blood routine, C-reactive protein (CRP), liver function, renal function, coagulation function, electrolytes, glycosylated hemoglobin, and fasting blood glucose levels of all patients were determined. The ACS was divided into 3 distinct groups: STEMI, NSTEMI, and UA. Further investigation was conducted into the factors influencing the inhibitory effect of SAA on platelets. The study protocol and all procedures were approved by the Ethics Committee of the Lishui Central Hospital, and all participants were given their written informed consent.

### 2.2. Chemical and drug

SAA (purity > 98%) was purchased from Plant Bio-Engineering Co. Ltd. (Xi’an, China) and dissolved in physiological saline (0.9% NaCl) to prepare a stock solution. The final experimental concentration was 0.1 mg/ml. Anti-CD62p/PE and platelet activation compound-1 (PAC-1)/FITC were obtained from Becton Dickinson (San Jose, CA). All chemicals and reagents were of analytical grade or the highest commercial quality.

### 2.3. Platelet aggregation

The maximum platelet aggregation induced by agonists was assessed using light transmittance aggregation (AggRAM, Helena Laboratories Inc., Beaumont, TX). Platelet-rich plasma (PRP) was obtained by centrifuging citrate-anticoagulated whole blood at 167g for 10 minutes at room temperature. The remaining sample was re-centrifuged at 2683 g for 5 minutes to obtain platelet-poor plasma. The samples were then preincubated with either physiological saline or SAA (final concentration: 0.1 mg/ml, dissolved in physiological saline) at 37°C without stirring for 10 minutes. We induced platelet aggregation with ADP (4 μM) or thrombin (0.4 U/ml), then recorded optical density for 5 minutes during aggregation. The aggregated levels were expressed as a percentage of the maximal light transmittance at baseline, where maximal transmittance represents platelet-poor plasma.

### 2.4. Flow cytometry

Platelet surface marker expression in PRP was analyzed using a Beckman Coulter EPICS XL flow cytometer (Beckman Coulter, Hialeah, FL). Briefly, 5 μL of PRP was incubated with 45 μL of phosphate-buffered saline containing fluorophore-conjugated monoclonal antibodies against PAC-1 (fibrinogen [FIB] receptor) and CD62p (P-selectin), each at a 1:20 dilution to minimize plasma interference. Samples were preincubated for 10 minutes at room temperature with either physiological saline (vehicle control) or SAA (final concentration: 0.1 mg/mL in saline). Subsequently, both groups were stimulated with agonists – either ADP (final concentration: 20 μM, standard for platelet activation studies) or thrombin (0.2 U/mL). After 15 minutes of incubation in the dark at room temperature, samples were fixed with 0.1% (v/v) formaldehyde in saline. Platelet populations were gated based on forward and side scatter (FSC/SSC), with PAC-1 binding quantified as the percentage of FIB-binding-positive platelets, and CD62p expression as the percentage of CD62p-positive platelets.

### 2.5. Statistical analysis

The data management and statistical analysis were carried out using IBM SPSS Statistics 26. The qualitative data were described using n (%) and then compared between groups using chi-square and Fisher exact tests. The results for quantitative data with a normal distribution were presented as mean + standard deviation (X + SD), with *t* tests applied for comparisons between groups. Mann–Whitney *U* tests were used to analyze differences between groups with non-normally distributed quantitative data. The correlation coefficients of Spearman and Pearson were calculated to explore the relationships between variables. Each test was set at a 0.05 significance level unless otherwise specified.

## 3. Results

### 3.1. Study population and clinical characteristics

This study included a total of 40 participants diagnosed with ACS, consisting of 29 males and 11 females, with an average age of 62.48 ± 9.22 years. A total of 19 patients (47.5%) and 7 patients (17.5%) had a smoking and alcohol history, respectively. The majority of patients, 37 (92.5%), were treated with dual antiplatelet therapy, and 31 (77.5%) received anticoagulant therapy. A total of 21 patients (52.5%) were diagnosed with hypertension, and 12 patients (30%) were diagnosed with diabetes mellitus. Table 1 provides a detailed description of the clinical characteristics of the patients.

**Table 1:** Anthropometric and biochemical characteristics of the subjects included in the study.

Variable	ACS (n = 40)
Gender: male (%)	29 (72.50)
Age (yr)	62.48 ± 9.22
BMI (kg/m^2^)	24.22 ± 3.68
Diabetics (%)	12 (30.00)
Hypertension (%)	21 (52.50)
Smoke (%)	19 (47.50)
Alcohol (%)	7 (17.50)
DAPT (%)	37 (92.50)
Anticoagulant (%)	31 (77.50)
PLT (×10^9^/L)	206.25 ± 45.22
PDW (%)	16.04 ± 1.52
MPV (fL)	9.81 ± 1.96
TC (mmol/L)	3.99 ± 0.87
LDL (mmol/L)	2.07 ± 0.62

The results are presented as mean ± SD or n (%).

ACS = acute coronary syndrome, BMI = body mass index, DAPT = dual antiplatelet therapy, LDL = low-density lipoprotein, MPV = mean platelet volume, PDW = platelet distribution width, PLT = platelet count, TC = total cholesterol.

### 3.2. Effects of SAA on platelet aggregation

**Figure 1.**
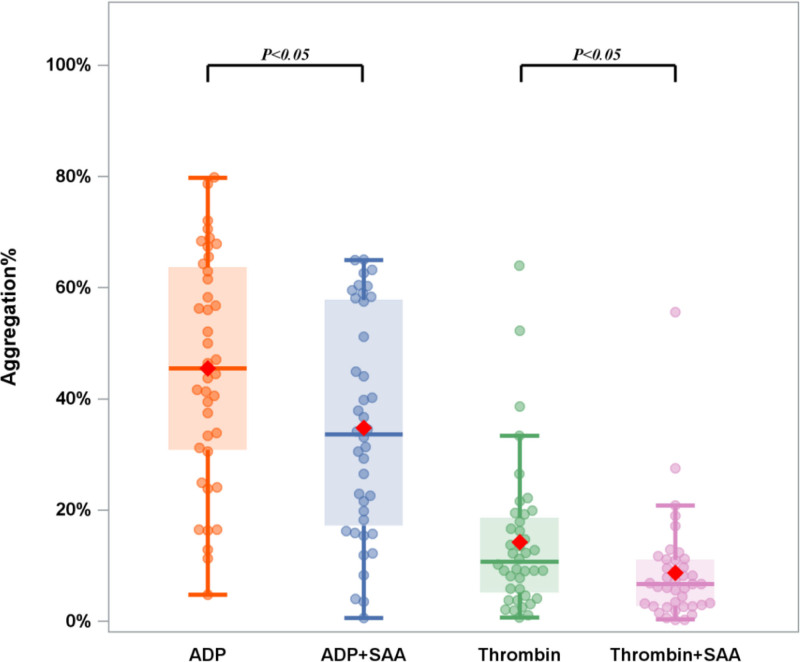
Effects of salvianolic acid A (SAA) on platelet aggregation. Platelet-rich plasma (PRP) was preincubated for 10 minutes with SAA (0.1 mg/mL) or a vehicle. Platelet aggregation was initiated with ADP (4 μM) or thrombin (0.4 U/mL). Data are presented as median (interquartile range, IQR). Error bars indicate IQR, and the horizontal line within the box represents the median. ADP = adenosine diphosphate, SAA = salvianolic acid A.

It was found that the mean maximum platelet aggregation rate was 45.45 (30.85, 63.65) % in the ADP group, 33.60 (17.20, 57.80) % in the (ADP + SAA) group, 10.07 (5.20, 18.55) % in the thrombin group and 6.70 (2.80, 11.05) % in the (thrombin + SAA) group. The study population was more susceptible to ADP-induced platelet aggregation than those exposed to thrombin (0.4 U/mL). Furthermore, our results indicated that ADP and preincubation with SAA (0.1 mg/mL) inhibited thrombin-induced aggregation. Taken together, these findings indicated that SAA inhibited the aggregation of platelets induced by ADP and thrombin (Fig. 1).

### 3.3. Impacts of SAA on agonist-induced single platelet activation

**Figure 2.**
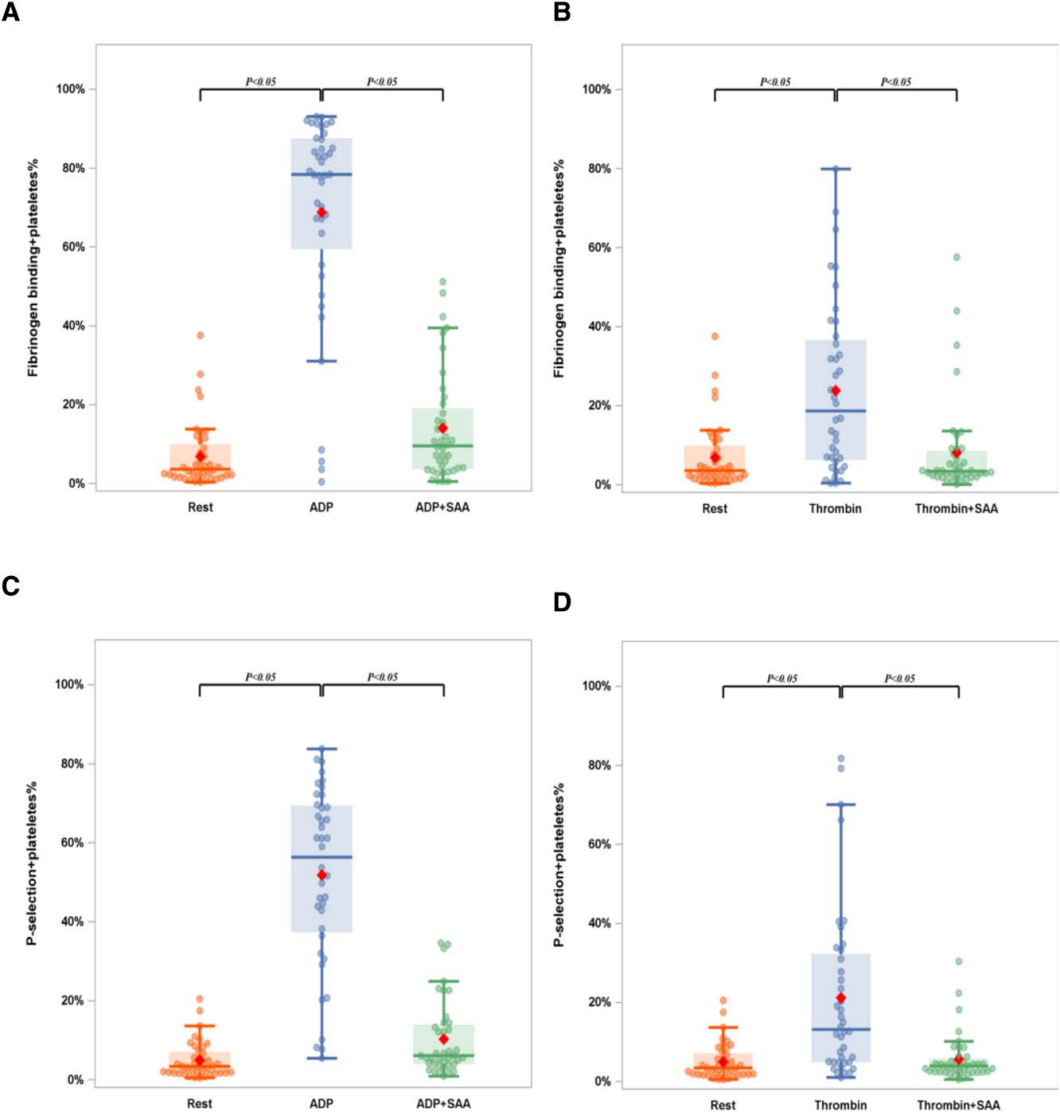
Impacts of SAA on single-platelet activation. Expression of (A) PAC-1 and (B) CD62p on platelets stimulated by ADP (20 μM) or thrombin (0.2 U/mL). Data are presented as median IQR. Error bars indicate IQR, and the horizontal line within the box represents the median. ADP = adenosine diphosphate, CD62p = P-selectin, PAC-1 = platelet activation compound-1, SAA = salvianolic acid A.

**Figure 3.**
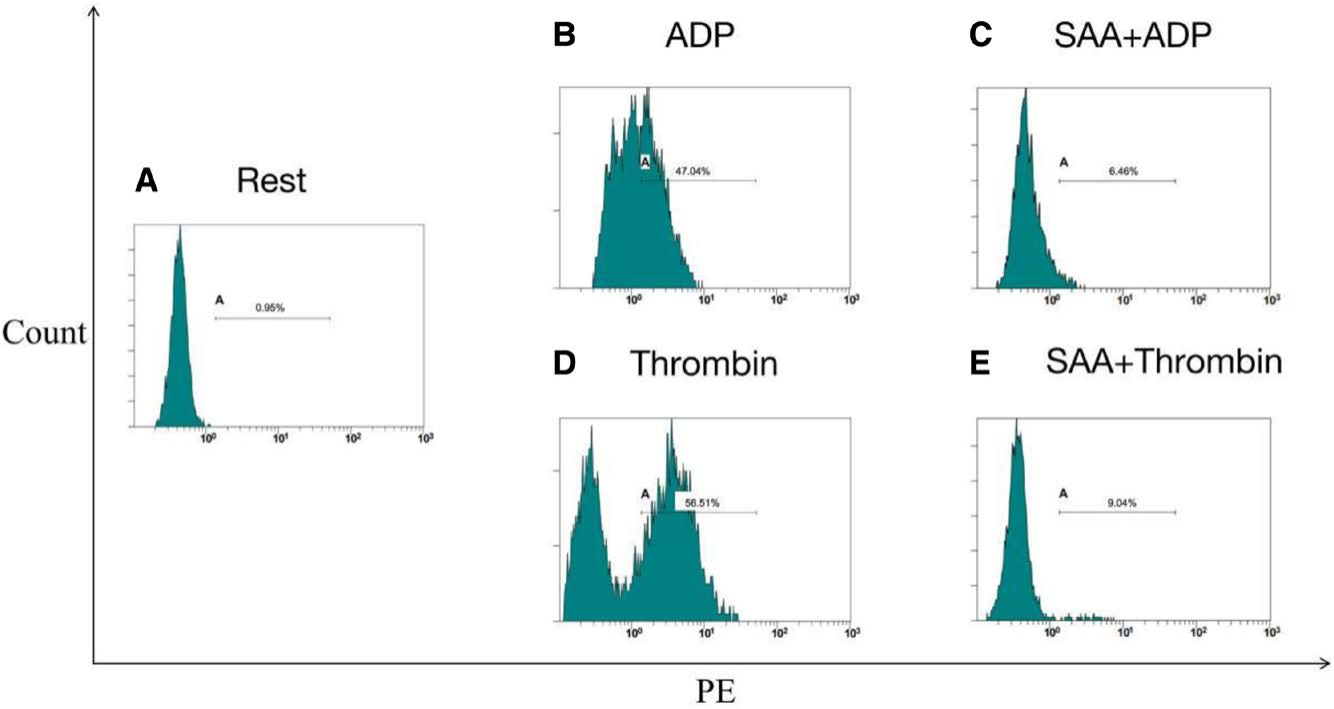
Representative histograms of the expression level of the active form of CD62p on non-stimulated platelets (shown as Rest). CD62p = P-selectin.

**Figure 4.**
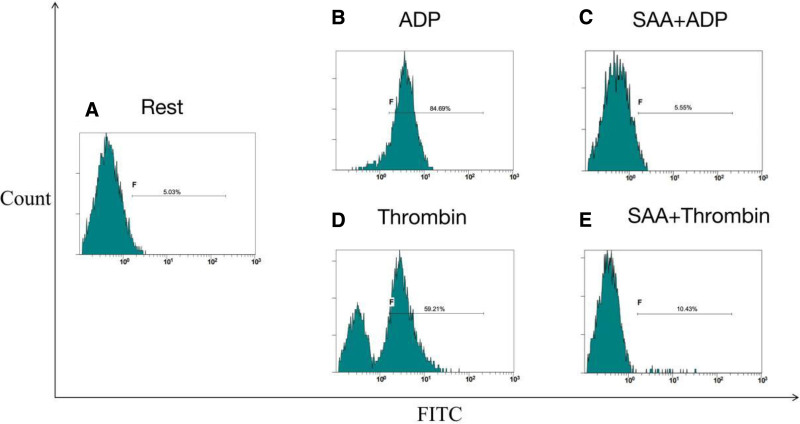
Representative histograms of the expression level of the active form of PAC-1 on non-stimulated platelets (shown as Rest). PAC-1 = platelet activation compound-1.

Flow cytometry was used to further explore the effects of SAA on single platelet activation in PRP. The results exhibited that ADP (20 μM) and thrombin (0.2 U/ml) elevated the expression of the PAC-1 aggregation marker from 3.61 (1.79, 9.89) % to 78.42 (59.48, 87.49) % and 18.69 (6.26, 36.56) %, respectively (all P 0.05). We found that SAA treatment reduced these increases to 9.58 (3.71, 18.99) % and 3.47 (2.15, 8.47) %, respectively. Similarly, the results also showed that ADP (20 μM) and thrombin (0.2 U/ml) significantly elevated the expression of CD62p from a baseline of 3.42 (1.75, 7.05) % to 56.36 (37.34, 69.33) % and 13.17 (4.88, 32.16) %, respectively. Moreover, our results revealed that Incubation with 0.1 mg/ml SAA reduced these increases to 6.13 (4.06, 13.89) % and 3.94 (2.44, 5.68) %, respectively. Taken together, the above findings revealed that SAA inhibited the induction of CD62p and PAC-1 by ADP and thrombin (Figs. 2–4).

### 3.4. Comparative antiplatelet effects of SAA across ACS subtypes

**Figure 5.**
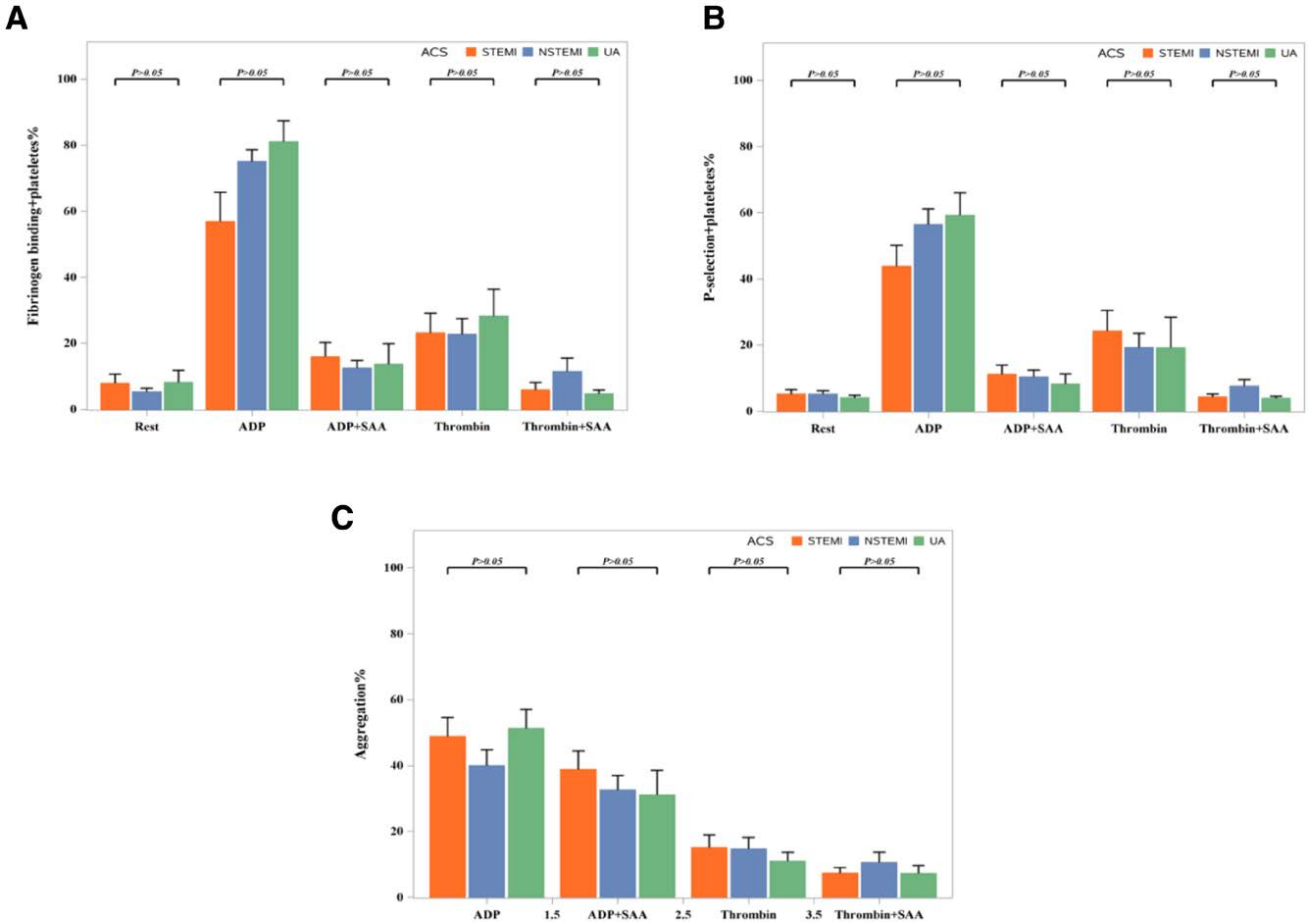
Antiplatelet effect of SAA among 3 types of ACS. ACS = acute coronary syndrome, SAA = salvianolic acid A.

In this study, 40 patients with ACS were included, 16 of whom were diagnosed with STEMI, 17 with NSTEMI, and 7 with UA. There were no statistically significant differences (*P* > .05) between the 3 groups in age, sex, body mass index, diabetes history, smoking history, alcohol consumption, dual antiplatelet therapy, anticoagulation therapy, CRP levels, CRP-to-albumin ratio (CAR), neutrophil percentage, hemoglobin levels, platelet-related indices, lipid-related indices and calcium ion levels. The prevalence of hypertension history was higher in the STEMI group compared to the NSTEMI group (68.75% vs 23.53%, *P* < .05). The clinical and biochemical baseline characteristics of the study population are summarized in Table 2. The levels of creatine kinase, troponin, aspartate aminotransferase, alanine aminotransferase (ALT), and albumin also differed significantly between the 3 groups. As shown in Figure 5, platelet aggregation was significantly higher in STEMI, NSTEMI, and UA patients under ADP induction. The results showed that preincubation with SAA efficaciously inhibited ADP-induced aggregation. Under thrombin stimulation, no significant differences were observed in platelet aggregation between the 3 groups. More importantly, our results showed that SAA significantly attenuated ADP and the expression of thrombin-induced CD62p and PAC-1 in patients in all 3 groups. Notably, SAA significantly attenuated ADP and thrombin-induced CD62p and PAC-1 expression in all ACS patients, suggesting a class effect independent of ACS subtypes.

**Table 2:** Baseline demographic data and clinical characteristics of the 3 groups included in the study.

Variable	STEMI (n = 16)	NSTEMI (n = 17)	UA (n = 7)
Age (yr)	60.5 ± 11.0	64.1 ± 8.7	63.1 ± 5.5
BMI (kg/m^2^)	24.8 ± 4.1	24.1 ± 3.9	23.1 ± 1.7
Gender: male (%)	14 (87.50)	11 (64.71)	4 (57.14)
Diabetics (%)	3 (18.75)	6 (35.29)	3 (42.86)
Hypertension (%)	11 (68.75)	4 (23.53)	6 (85.71)*
Smoke (%)	9 (56.25)	6 (35.29)	4 (57.14)
Alcohol (%)	3 (18.75)	2 (11.76)	2 (28.57)
DAPT (%)	15 (93.75)	16 (94.12)	6 (85.71)
Anticoagulant (%)	15 (93.75)	12 (70.59)	4 (57.14)
CRP (mg/L)	11.67 (2.57–42.06)	2.37 (1.29–15.24)	2.60 (1.30–4.60)
WBC (×10^9^/L)	11.93 ± 4.69	8.22 ± 3.13	7.97 ± 3.06*
NEUT (%)	74.98 ± 11.29	75.73 ± 8.90	71.06 ± 10.47
Hb (g/L)	146.00 (123.00–153.00)	139.00 (131.00–150.00)	121.00 (115.00–137.00)
PLT (×10^9^/L)	214.19 ± 41.36	197.76 ± 52.95	208.71 ± 34.08
MPV (fL)	10.05 (9.70–10.25)	9.40 (9.00–10.70)	10.10 (10.00–10.70)
PDW (%)	16.40 (16.15–16.55)	16.30 (15.90–16.70)	16.40 (15.90–16.50)
CK (U/L)	57.35 (15.01–126.50)	7.49 (2.20–16.10)	1.02 (0.23–4.32)*
Myo (ng/ml)	164.15 (75.10–564.60)	49.20 (38.78–284.50)	38.70 (34.00–122.00)
Tn (ng/ml)	30.05 (4.21–69.00)	2.14 (0.88–6.25)	0.05 (0.04–0.12)*
ALT (U/L)	51.00 (29.00–66.00)	22.00 (11.00–37.00)	24.00 (18.00–41.00)*
AST (U/L)	128.00 (50.50–225.00)	27.00 (19.00–50.00)	23.00 (20.00–56.00)*
Alb (g/L)	33.85 ± 5.02	36.28 ± 4.11	39.47 ± 3.06*
CAR	0.35 (0.08–1.16)	0.06 (0.03–0.45)	0.07 (0.03–0.12)
eGFR	94.40 ± 19.67	73.12 ± 33.23	86.26 ± 19.17
GLU (mmol/L)	6.02 (5.11–7.82)	6.31 (5.20–7.57)	5.79 (5.34–6.32)
TC (mmol/L)	3.97 ± 0.88	4.10 ± 0.97	3.79 ± 0.56
TG (mmol/L)	1.44 (1.16–1.69)	1.24 (1.06–2.10)	1.23 (1.00–1.68)
HDL (mmol/L)	0.94 (0.85–1.07)	1.16 (0.97–1.29)	1.12 (0.99–1.22)
LDL (mmol/L)	2.17 ± 0.63	2.05 ± 0.70	1.91 ± 0.38
CA^2+^ (mmol/L)	2.14 ± 0.10	2.17 ± 0.13	2.24 ± 0.13

The results are presented as mean ± SD or n (%).

Alb = albumin, ALT = alanine aminotransferase, AST = aspartate aminotransferase, BMI = body mass index, CAR = C-reactive protein-to-albumin ratio, CK = creatine kinase, CRP = C-reactive protein, eGFR = estimated glomerular filtration rate, GLU = glucose, Hb = hemoglobin, HDL = high-density lipoprotein, LDL = low-density lipoprotein, MPV = mean platelet volume, Myo = myoglobin, NSTEMI = non-ST-elevated myocardial infarction, PDW = platelet distribution width, PLT = platelet count, STEMI = ST-elevated myocardial infarction, TC = total cholesterol, TG = triglyceride, Tn = troponin, UA = unstable angina pectoris, WBC = white blood cell count.

* *P* < .05.

### 3.5. Analysis of factors related to the antiplatelet effect of SAA

In this study, we assessed the factors affecting the platelet inhibitory effect of SAA by measuring the difference in CD62P expression percentage between the ADP group and (ADP + SAA) group, which we set as dif1. An exhaustive single-factor analysis of various clinical variables identified pivotal factors affecting SAA platelet inhibitory effects, as shown in Table 3. Statistical differences between the platelet inhibitory effect of SAA and sex, diabetes, hypertension, alcohol consumption, anticoagulant medications, and ACS were insignificant for mean dif1, suggesting that these factors may not have a considerable effect. The mean value of dif1 showed a substantial difference between the smoking and nonsmoking cohorts (*P* = .005), suggesting that smoking was a critical factor in SAA platelet inhibition efficacy in patients with ACS.

**Table 3:** Single factor analysis.

Variable	Group	n (%)	Description	*χ*2/*t*/*Z*	*P* value
Gender	Male	29 (72.5)	37.61 ± 21.17	1.81	.078
	Female	11 (27.5)	51.86 ± 24.79		
Diabetics	No	28 (70.0)	40.77 ± 25.96	0.32	.753
	Yes	12 (30.0)	43.30 ± 13.67		
Hypertension	No	19 (47.5)	40.11 ± 22.90	0.37	.713
	Yes	21 (52.5)	42.81 ± 23.24		
Smoke	No	21 (52.5)	50.91 ± 21.19	3.00	.005*
	Yes	19 (47.5)	31.16 ± 20.33		
Alcohol	No	33 (82.5)	43.60 ± 21.07	1.26	.216
	Yes	7 (17.5)	31.74 ± 29.68		
DAPT	No	3 (7.5)	16.65 ± 33.21	2.04	.048*
	Yes	37 (92.5)	43.54 ± 21.15		
Anticoagulant	No	9 (22.5)	43.01 ± 27.54	0.22	.827
	Yes	31 (77.5)	41.09 ± 21.77		
ACS	STEMI	16 (40.0)	32.53 ± 24.96	1.53	.111
	NSTEMI	17 (42.5)	46.10 ± 18.31		
	UA	7 (17.5)	50.98 ± 23.54		

ACS = acute coronary syndrome, DAPT = dual antiplatelet therapy, NSTEMI = non-ST-elevated myocardial infarction, STEMI = ST-elevated myocardial infarction, UA = unstable angina pectoris.

* *P* < .05.

**Figure 6.**
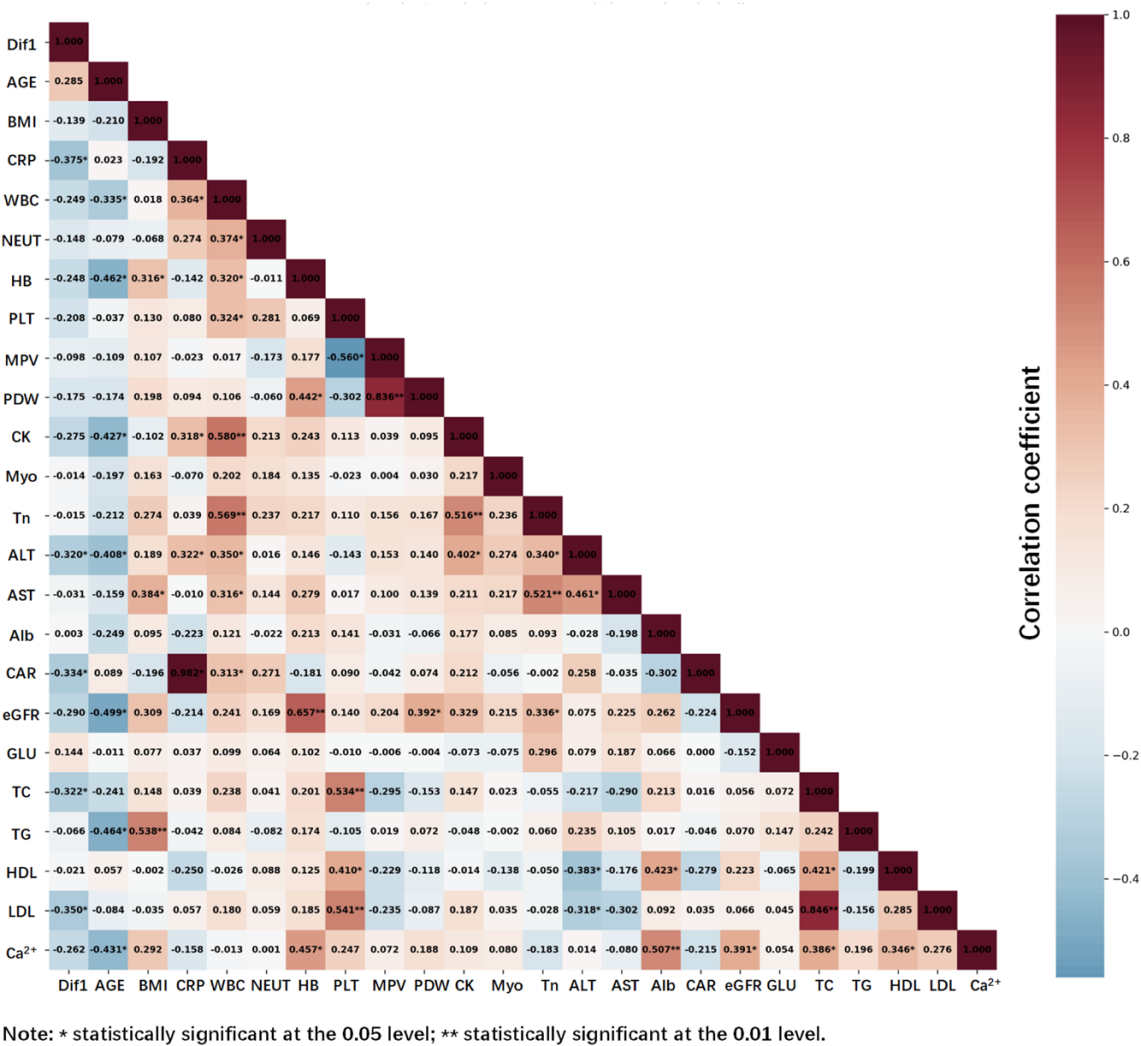
Correlation of multiple variables with dif1.

The multiple linear regression model was fitted using the stepwise method (Table 4). There is no multicollinearity between variables based on the variable variance inflation factor. The results indicated that smoking, CAR, ALT, and low-density lipoprotein (LDL) were the main factors contributing to the difference. Higher CAR, ALT, and LDL levels result in a lower difference. The correlation heatmap shows that dif1 strongly correlates with CRP, ALT, CAR, total cholesterol, and LDL, indicating a significant correlation between dif1 and the above variables (Fig. 6).

**Table 4:** Multiple linear regression.

Variable	Nonstandardized regression coefficient		*β*	*t*	*P* value	VIF
	*β*	Standard errors				
Intercept	88.751	12.157		7.300	<.001	0.000
smoke	−16.25	6.049	−0.360	−2.686	.011	1.203
CAR	−3.684	1.604	−0.303	−2.297	.028	1.169
ALT	−0.247	0.121	−0.291	−2.044	.049	1.357
LDL	−13.13	5.022	−0.358	−2.615	.013	1.257

ALT = alanine aminotransferase, CAR = C-reactive protein-to-albumin ratio, LDL = low-density lipoprotein, VIF = variance inflation factor.

## 4. Discussion

This study evaluated the inhibitory effects of SAA on platelet activation and aggregation in ACS patients in detail. It confirmed that SAA could significantly reduce platelet activity induced by various stimulants by flow cytometry and platelet aggregation detection techniques. SAA had a similar antiplatelet effect on all 3 types of ACS (UA, NSTEMI, and STEMI), which provided a broad range of clinical applications.

The membrane glycoprotein CD62P is expressed in the granular membrane of platelets.^[13]^ Platelets secrete a granular membrane, which fuses with the platelet cell membrane and increases CD62P expression.^[14]^ Thus, CD62P is a marker of platelet secretion.^[15]^ The FIB-binding protein PAC-1 is also a glycoprotein on the platelet membrane.^[11]^ The GPIIb/IIIa receptor on the platelet surface undergoes conformational changes after activation.^[16]^ PAC-1 represents platelet aggregation capacity, and the 2 above indicate platelet activation.^[17]^

ACS is caused by the activation and aggregation of platelets, which is one of the leading causes of coronary thrombosis. Activated platelets release pro-coagulant factors and express adhesion molecules,^[18]^ promote the aggregation of platelets and increase the formation and stability of thrombosis, resulting in myocardial ischemia and necrosis.^[4]^ In this present study, the activation and aggregation of platelets were measured by flow cytometry and photometry. We found that the expression of CD62P and PAC-1 was significantly increased under thrombin and ADP stimulation, as was the maximum platelet aggregation rate. Our results also demonstrated that pre-incubation of SAA downregulated the expression of CD62P and PAC-1 and maximum platelet aggregation.

The thrombin flow cytometry histogram showed 2 peaks during the detection of platelet activation, while the ADP flow cytometry histogram only showed 1 peak. This may be related to the complex process of thrombin-platelet activation, as reported in previous studies. A growing body of evidence indicates that thrombin triggers the activation of various platelet subsets through regulating the PRA1 and PRA4 receptors.^[19–21]^ Studies have revealed that platelets can be divided into hyperactivated and activated subgroups after stimulation with stimulants.^[22]^ Hyperactivated platelets showed high factor V binding and high phosphatidylserine exposure. Alternatively, the activated subgroup exhibited relatively low levels of factor V binding and phosphatidylserine exposure.^[21]^ Thrombin groups show 2 peaks due to successive activation of the subgroups. The results of the pre-incubation of SAA suggest that SAA effectively inhibits the multi-stage activation of platelets induced by thrombin. ADP stimulates calcium release from platelets and activates surface integrins through the P2Y1 and P2Y12 receptors.^[23]^ Therefore, platelets appear as a single peak of activation.

In our study, the majority of patients received antiplatelet and anticoagulant therapy at the primary level, which demonstrates the success of the construction of the Chest Pain Center in China. In addition to existing therapies, SAA can reduce platelet activity. SAA inhibited platelet activation induced by multiple agonists, which suggests that it may have broad application potential, especially for patients with ACS with a high level of platelet activity. Previous studies have revealed that SAA inhibits cell growth by regulating multiple signaling pathways.^[12]^ Recent studies have shown that SAA inhibits platelet aggregation by impeding the PI3K/Akt signaling pathway, phosphorylating Akt (Ser 473/474), and impeding Rap1 activation.^[10]^ In addition, studies have shown that SAA exerts antiplatelet properties by increasing the cyclic adenosine monophosphate level. Cyclic adenosine monophosphate, as an important intracellular signaling molecule, inhibits platelet activation by activating adenylate cyclase. SAA provides a unique advantage over traditional antiplatelet agents such as aspirin, clopidogrel, and GPIIb/IIIa receptor antagonists.^[24]^ Researchers have also observed that SAA promotes the function of platelets without impacting coagulation function and does not affect prothrombin time, activated partial thromboplastin time, FIB, and thrombin time in normal rats.^[25]^ Several lines of studies have revealed that traditional antiplatelet drugs also increase bleeding risks in patients when they exert their antiplatelet effects.^[26,27]^ In contrast, SAA exerts antiplatelet effects without significantly interfering with the clotting process, suggesting that it has a superior safety profile and might be an ideal treatment option for patients with bleeding complications.

This present study explored the influencing factors of SAA inhibiting platelet activity in ACS patients and found that smoking, inflammation, LDL, and ALT factors might affect the antiplatelet effect of SAA. SAA cannot significantly inhibit platelet activity in this population based on their high CARs, ALTs, and LDLs. Growing evidence indicates that smoking may promote platelet activation by the activation of oxidative stress^[28]^ and reduce the inhibitory effect of SAA on platelet activity.^[29]^ Inflammation is associated with platelet reactivity.^[30]^ Platelets release several inflammatory factors to promote vascular permeability during inflammation.^[31]^ SAA reduces the antiplatelet effects of platelets by increasing their reactivity. This study used CAR instead of CRP as an inflammation indicator. Accumulating evidence indicates that CAR can provide a more comprehensive representation of inflammation within the body during acute events.^[32,33]^ In addition, previous studies have revealed that ALT levels and platelet activity are associated when liver cells are damaged or disease occurs, resulting in elevated serum ALT levels.^[34]^ Impaired liver function can disrupt the normal function of the clotting system, inducing platelets to become overactive. Increasing evidence suggests that LDL can be oxidized and endocytosed by macrophages and can form cholesterol-rich plaques that promote adhesion, aggregation, and secretion of platelets.^[31,35]^ Increased LDL and ALT levels may also affect the effect of SAA by altering the fluidity or activity of the platelet membrane. The results of our current study are consistent with those of previous studies. Several clinical implications can be drawn from these findings, particularly in the development of individualized treatment programs and health education.^[36]^ Additionally, we must consider the limitations of our studies, such as the sample specificity and other interfering factors. There was a limited number of patients enrolled in the study, and the majority of those included were receiving anticoagulation and antiplatelet therapy, preventing a comprehensive evaluation of the antiplatelet effect of SAA on patients with ACS. In addition, patients treated with SAA cannot be measured for platelet reactivity since there is insufficient evidence to suggest that SAA can be safely administered to humans. Future research directions may include exploring whether these factors affect the efficacy of currently available antiplatelet medications, investigating the molecular mechanisms underlying the effects of smoking, CAR, ALT, and LDL on SAA, and exploring ways to improve the effectiveness of SAA.

## 5. Conclusion

In this study, the antiplatelet effect of SAA in patients with ACS was evaluated using flow cytometry and platelet aggregation techniques. More importantly, the results indicated that SAA inhibited platelet aggregation and activation in patients with ACS, and these inhibitory effects were not different among patient types. Therefore, our findings suggest that SAA has the potential to be a novel and effective antiplatelet drug for the treatment of ACS.

## Author contributions

**Conceptualization:** Peipei Wang, Chunlai Zeng.

**Data curation:** Peipei Wang, Shunqiong Zhang, Abdullah A.I. Mamun.

**Formal analysis:** Shuanglin Xie.

**Funding acquisition:** Peipei Wang.

**Investigation:** Peipei Wang, Shunqiong Zhang, Xinyuan Li, Wei Zhang, Abdullah A.I. Mamun, Wenjuan Lu.

**Methodology:** Peipei Wang.

**Resources:** Chunlai Zeng.

**Software:** Peipei Wang, Shunqiong Zhang, Xinyuan Li, Shuanglin Xie, Yibin Mei, Wei Zhang.

**Supervision:** Chunlai Zeng.

**Validation:** Peipei Wang, Shunqiong Zhang, Xinyuan Li, Yibin Mei, Taoqing Liang.

**Visualization:** Peipei Wang, Shuanglin Xie.

**Writing – original draft:** Peipei Wang.

**Writing – review & editing:** Peipei Wang, Chunlai Zeng.
